# Supplementary material for: Comparative Metabolomic Analysis of Moringa oleifera Leaves of Different Geographical Origins and Their Antioxidant Effects on C2C12 Myotubes
Source: Int J Mol Sci. 2024 Jul 25;25(15):8109. doi: 10.3390/ijms25158109 (PMC11311983; doi:10.3390/ijms25158109)
Supplement: Supplementary file 1 [file ijms-25-08109-s001.zip › ijms-3059249-supplementary.pdf]

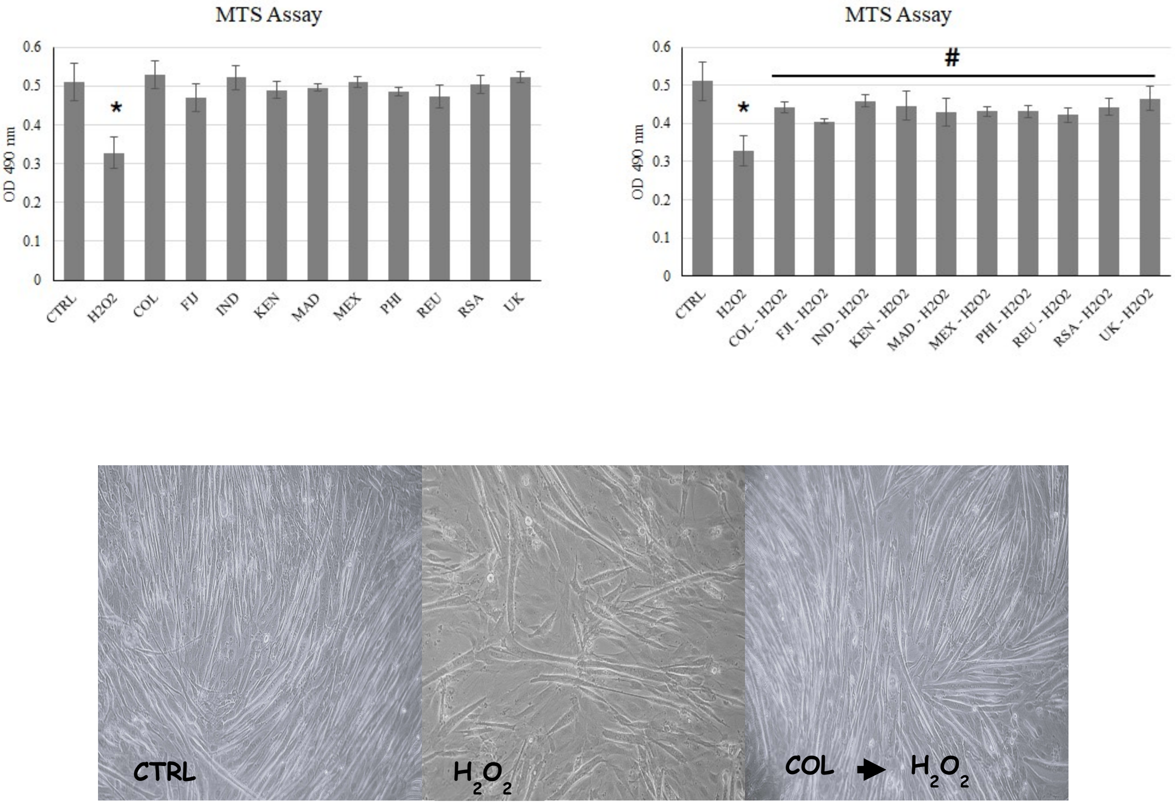


**Supplementary material figure S1.** MTS assay. (**Upper Panel left**): C2C12 myotubes were treated with various dilutions of MOLE stock solution (1/100 working solution) or vehicle (ethanol) in culture media for 24 hours. During the final hour of treatment, a sample treated solely with 1 mM $H_2O_2$ was tested. (**Upper Panel right**): C2C12 myotubes were treated with MOLE or vehicle (ethanol) in culture media for 24 hours. Hydrogen peroxide (1 mM) was then added to the MOLE pre-treated samples for an additional hour. Cell viability was assessed using the MTS assay.
(**Lower Panel**): C2C12 Myotubes representative images.
Data are presented as the mean ± S.D. of three experiments, each performed in triplicate. *$p < 0.01$ vs. CTRL; #$p < 0.05$ vs. $H_2O_2$.
